# Visualization and clinical relevance of the endolymphatic duct and sac in Ménière's disease

**DOI:** 10.3389/fneur.2023.1239422

**Published:** 2023-08-31

**Authors:** Lisa M. H. de Pont, Maartje T. P. M. Houben, Thijs O. Verhagen, Berit M. Verbist, Mark A. van Buchem, Claire C. Bommeljé, Henk M. Blom, Sebastiaan Hammer

**Affiliations:** ^1^Department of Radiology, Haga Teaching Hospital, The Hague, Netherlands; ^2^Department of Radiology, Leiden University Medical Center, Leiden, Netherlands; ^3^Department of Otorhinolaryngology, Haga Teaching Hospital, The Hague, Netherlands; ^4^Department of Otorhinolaryngology, Leiden University Medical Center, Leiden, Netherlands; ^5^Department of Otorhinolaryngology, Antwerp University Hospital, Antwerp, Belgium

**Keywords:** Ménière, endolymphatic duct, endolymphatic sac, MRI, clinical features

## Abstract

**Background:**

Ménière's disease (MD) is a chronic inner ear disorder with a multifactorial etiology. Decreased visualization of the endolymphatic duct (ED) and sac (ES) is thought to be associated with MD, although controversy exists about whether this finding is specific to MD. Recent literature has revealed that two distinct ES pathologies, developmental hypoplasia and epithelial degeneration, can be distinguished in MD using the angular trajectory of the vestibular aqueduct (ATVA) or ED-ES system as a radiographic surrogate marker. It has been suggested that these two subtypes are associated with distinct phenotypical features. However, the clinical differences between the ATVA subtypes require further validation.

**Research objective:**

The objective of this study is to investigate whether (1) non-visualization of the ED-ES system is a discriminative radiological feature for MD in a cohort of vertigo-associated pathologies (VAPs) and whether (2) different angular trajectories of the ED-ES system in MD are associated with distinguishable clinical features.

**Setting:**

The study was conducted in the Vertigo Referral Center (Haga Teaching Hospital, The Hague, the Netherlands).

**Methods:**

We retrospectively assessed 301 patients (187 definite MD and 114 other VAPs) that underwent 4h-delayed 3D FLAIR MRI. We evaluated (1) the visibility of the ED-ES system between MD and other VAP patients and (2) measured the angular trajectory of the ED-ES system. MD patients were stratified based on the angular measurements into α_exit_ ≤ 120° (MD-120), α_exit_ 120°-140° (MD-intermediate), or α_exit_ ≥ 140° (MD-140). Correlations between ATVA subgroups and clinical parameters were evaluated.

**Results:**

Non-visualization of the ED-ES system was more common in definite MD patients compared with other VAPs (*P* < 0.001). Among definite MD patients, the MD-140 subtype demonstrated a longer history of vertigo (*P* = 0.006), a higher prevalence of bilateral clinical disease (*P* = 0.005), and a trend toward a male preponderance (*p* = 0.053). No significant differences were found between ATVA subgroups regarding the presence or severity of auditory symptoms, or the frequency of vertigo attacks.

**Conclusion:**

Non-visualization of the ED-ES system is significantly associated with MD. Among MD patients with a visible ED-ES system, we demonstrated that the MD-140 subtype is associated with a longer disease duration, a higher prevalence of bilateral MD, and a trend toward a male preponderance.

## Introduction

Ménière's disease (MD) is a chronic condition affecting the inner ear, that is diagnosed clinically by a constellation of cochlear and vestibular symptoms (1, 2). The heterogeneity of MD poses a diagnostic and management challenge: the disease is widely variable regarding the age of onset, symptom manifestation, disease course, and the development of bilateral involvement (3, 4).

Despite extensive research, a thorough understanding of the pathophysiological processes in MD is currently lacking (5). Previous literature has supported genetic, autoimmune, and allergic factors as possible contributing factors (6). Endolymphatic hydrops (EH) remains at the heart of MD's pathophysiology since its first description on temporal bone studies in 1938, although controversy exists over its role in the disease process (7–9). Application of delayed gadolinium-enhanced inner ear MRI in living patients has established EH as a radiographic marker for MD, as well as increased perilymphatic enhancement, which is thought to reflect disruption of the blood–labyrinthine barrier (BLB) (10). Research indicates that the regulation of endolymph homeostasis is complex and likely dominated by ionic transport systems (11). The endolymphatic duct (ED) and the endolymphatic sac (ES) are non-sensory components of the membranous labyrinth and are believed to be involved in endolymph volume regulation (11, 12). The ED leads to the blind-ending ES through the vestibular aqueduct (VA), a bony canal extending from the medial wall of the vestibule to the posterior surface of the temporal bone. The ES is located partly within the VA (intra-osseous part) and partly between the layers of the dura mater in the posterior cranial fossa (extra-osseous part) (13). The VA can be visualized on computed tomography (CT), whereas the ED-ES system can be delineated on MRI (13–15).

Previous authors have explored morphological variations of the ED-ES system in MD patients that may theoretically act as precipitating factors to the formation of EH. The VA and ED-ES system have been reported to be significantly smaller or non-visible in MD patients compared with healthy controls (16–22). However, narrowing of the VA has also been described in other inner ear diseases, such as chronic infection, vertigo, and (sudden or progressive) sensorineural hearing loss (SSNHL) (23, 24). Some authors have therefore argued that non-visualization of the VA is a non-specific sign of temporal bone pathology rather than specific for MD (25).

Currently, MD is assumed to be a multifactorial condition, and increasing efforts are made to subtype MD patients based on specific etiologies (5, 26). In 2018, Eckhard et al. first described two etiologically different pathologies of the ES in human temporal bone studies that were almost consistently present in patients with idiopathic EH with or without clinical MD: epithelial degeneration and developmental hypoplasia (27). In 2019, Bächinger et al. published a method to distinguish these ES pathologies using the angular trajectory of the vestibular aqueduct (ATVA) as a radiographic surrogate marker. This method was initially developed for ATVA measurement on histological temporal bone specimens and thereafter adapted for high-resolution computed tomography (HRCT) and gadolinium-enhanced magnetic resonance imaging (Gd-enhanced MRI) in living patients (28, 29). The importance of their research is noted by the phenotypical differences they described between the two ES endotypes regarding age of onset, gender distribution, frequency of vertigo attacks, and bilateral involvement (27–30). The recognition of etiologically and clinically distinct subgroups within MD may lead to a better understanding of its pathophysiology, provide prognostic information, and can potentially aid in the development of personalized or tailored treatment strategies. However, the ATVA method of Bächinger et al. has not been widely applied and the phenotypical differences between the MD endotypes require further validation.

Therefore, this study aimed to investigate to what extent non-visualization of the ED-ES system on delayed Gd-enhanced FLAIR MRI is a feature of MD in a cohort of vertigo-associated pathologies, and, if visible, whether different angular trajectories of the ED-ES system in MD are characterized by distinguishable clinical features.

## Materials and methods

### Study population

**Figure 1:**
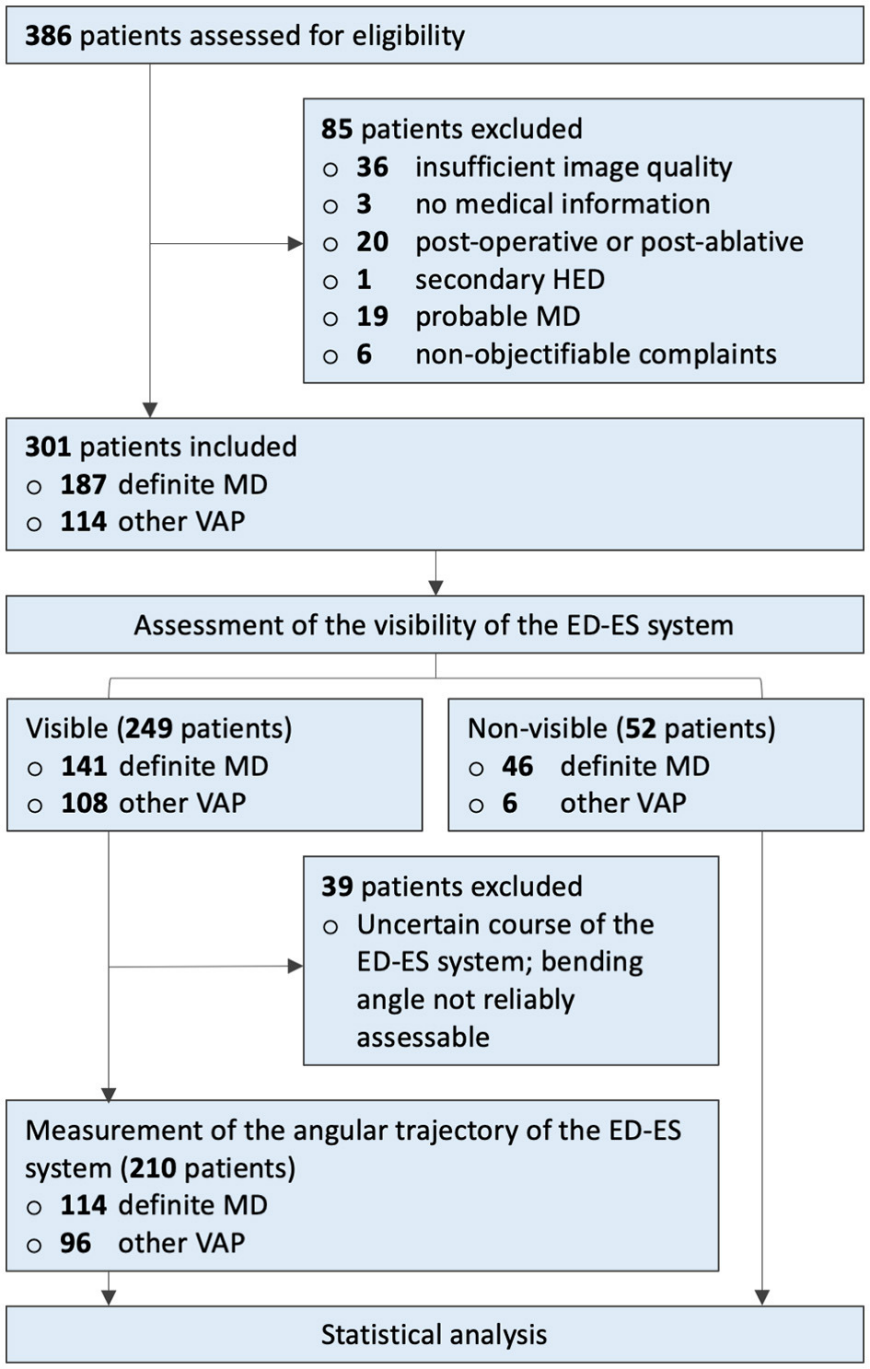
Inclusion flowchart. ATVA, angular trajectory of the vestibular aqueduct; ED, endolymphatic duct; ES, endolymphatic sac; Gd, gadolinium; HED, hydropic ear disease; MD, Ménière's disease; MRI, magnetic resonance imaging; VAP, vertigo-associated pathology.

We retrospectively assessed 386 consecutive patients presenting to our vertigo referral center (Haga Teaching Hospital, The Hague) with symptoms suspected of inner ear pathology, most commonly MD, that underwent 4 h-delayed Gd-enhanced MRI at our institution from February 2017 to March 2019. Patients with a technically inadequate MRI examination, unavailable medical data, previous temporal bone surgery, or intratympanic treatment with gentamycin prior to MRI were excluded, as well as those with secondary hydropic ear disease (HED) or a probable MD diagnosis. We also excluded six patients who were initially referred to our Otorhinolaryngology department with non-specific complaints, but whose symptoms could not be confirmed after clinical evaluation. Finally, 301 patients were statistically analyzed (Figure 1).

### MRI protocol

Imaging examinations were carried out on a 3T MRI scanner (Magnetom Skyra; Siemens, Erlangen, Germany) with a 20-channel array head coil, 4 h after intravenous gadolinium administration (30 mL gadoterate meglumine, Dotarem; Guerbet, Aulnay-sous-Bois, France). Patients were evaluated in the supine position with additional fixation between the patient's head and receiver coil to reduce motion artifacts. We acquired a three-dimensional fluid-attenuated inversion recovery (3D FLAIR) sequence with the following parameters: field of view, 190 mm; section thickness, 0.8 mm; repetition time, 6,000 ms; echo time, 177 ms; a number of excitations, 1; inversion time 2,000 ms; flip angle, 180°; matrix, 384 × 384; bandwidth, 213 Hz/pixel; turbo factor, 28; voxel size, 0.5 × 0.5 × 0.8 mm; acquisition time, 14 min. High-resolution T2 sampling perfection with application-optimized contrasts using different flip angle evolution (SPACE sequence; Siemens) images of the inner ear were obtained for anatomic reference of the entire labyrinthine fluid space. The scan parameters for the T2 SPACE sequence were as follows: field of view, 160 mm; section thickness, 0.5 mm; repetition time, 1,400 ms; echo time, 155 ms; number of excitations, 1; flip angle, 120°; matrix, 320 × 320; bandwidth, 289 Hz/pixel; turbo factor, 96; voxel size, 0.5 × 0.5 × 0.5; acquisition time, 5 min.

### Inner ear evaluation

#### Visualization of the ED-ES system

**Figure 2:**
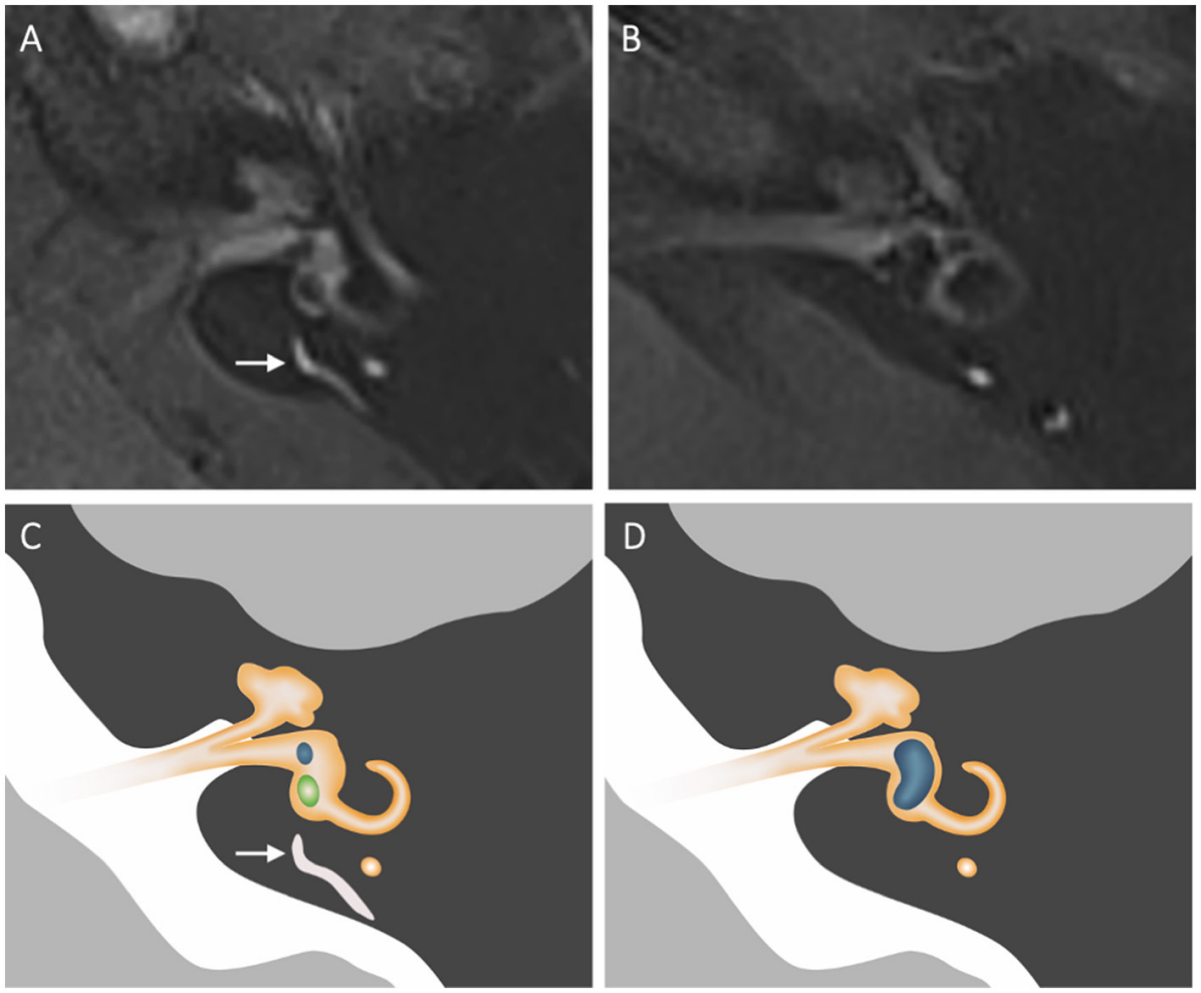
Morphology of the ED-ES complex on axial contrast-enhanced FLAIR images. The visibility of the ED-ES system was evaluated from the posteromedial aspect of the vestibule to the opercular region. **(A)** Normal visualization of the ED-ES system (long arrow) in a patient diagnosed with vestibular migraine. **(B)** Non-visualization of the ED-ES system in patient with definite MD. **(C, D)** Corresponding schematic depiction of the MR images. The visible parts of the cochlea, vestibule, and semicircular canals are depicted in orange. In **(C)**, the saccule and utricle are depicted in blue and green, respectively. Note the presence of vestibular hydrops (depicted in blue) in **(D)**.

The visibility of the ED-ES system was evaluated on a 4 h-delayed 3D FLAIR MRI. Visibility of the ED-ES refers to the visualization of either a clear linear or punctate region of enhancement in the expected course of the ED and ES from the posteromedial part of the vestibule through the vestibular aqueduct to the opercular region of the temporal bone, on more than one MRI section. Ears were stratified into either of two groups: visible ED-ES or non-visible ED-ES. Examples are presented in Figure 2.

#### Angular measurements and stratification of the ED-ES system

In total, 249 patients with a visible ED-ES system were evaluated for ATVA analysis. In 39 definite MD and other VAP patients, the ED-ES system was visible, but its angular trajectory could not be measured reliably due to either motion artifacts or an uncertain course of the ED-ES system in the opercular region, and these patients were excluded from further ATVA analysis (see inclusion chart, Figure 1). For the remaining 210 patients (114 definite MD and 96 other VAPs), angular measurements of the ED-ES system were performed by one PhD student (LP), who was blinded to the patient's clinical data, using the software provided by Bächinger et al. (28). This program provides a precustomized shape representing the vestibule and horizontal semicircular canal of the vestibular organ. The proximal trajectory of the ED at its origin from the vestibule is below the spatial resolution of current imaging techniques; to overcome this limitation, the software provides a red line I_1_ that is attached to the vestibular shape at a fixed angle of 14°, representing the average entrance angle (α_entrance_) of the ED in the temporal bone (as determined by a previous histopathological study in humans) (28). By fitting the shape into the boundaries of the corresponding anatomy and a second line parallel to the most distal part of the ED-ES system in the opercular region, the exit angle (α_exit_) is calculated.

**Figure 3:**
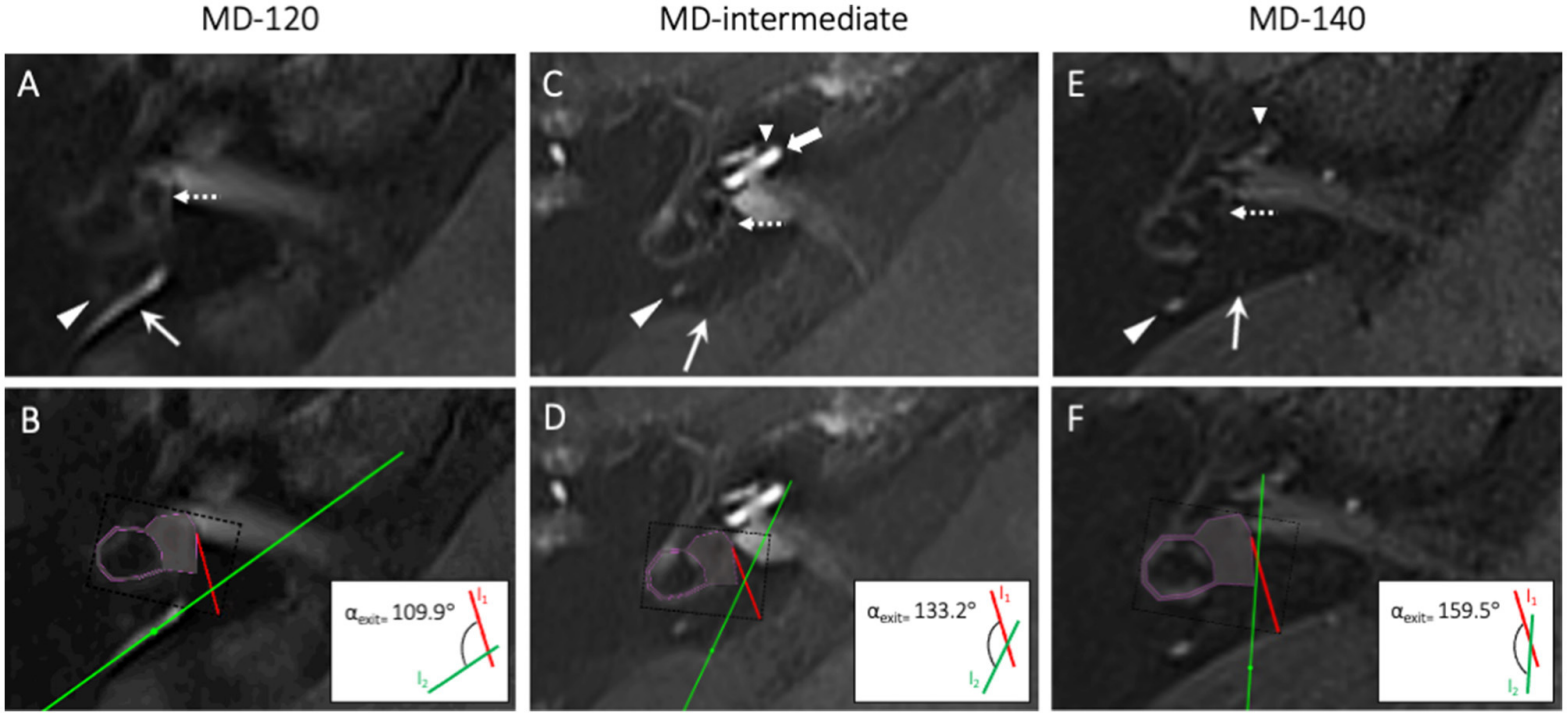
Angular trajectory of the ED-ES system in definite MD cases with MD-120 **(A, B)**, MD-intermediate **(C, D)**, and MD-140 **(E, F)** morphologies. **(A, C, E)** axial 4 h-delayed Gd-enhanced 3D FLAIR MRI, the long arrows indicate the ED-ES system in the opercular region. The ED-ES system lies in close proximity to the posterior semicircular canal (long arrow head). **(B, D, F)** show the corresponding ATVA by fitting a predefined shape (magenta shape) into the bony boundaries of the vestibule and horizontal semicircular canal. The red line (I_1_) is attached to this shape at a fixed angle of 14°, representing the entrance angle (α_entrance_) of the proximal ED in the temporal bone at its origin from the vestibule. The green line (I_2_) was fitted parallel to the trajectory along which the ES exits the temporal bone. The exit angle (α_exit_) of the ED-ES system was calculated by the software as the angle between I_1_ and I_2_. Note the presence of vestibular hydrops in all cases (dashed arrow in **A, C, E**), cochlear hydrops in the MD-intermediate and MD-140 cases (short arrowhead in **C, E**), as well as increased perilymphatic enhancement in the MD-intermediate case (thick arrow in **C**).

Following the classification proposed by Bächinger et al., 114 MD patients were stratified into α_exit_ ≤ 120° or α_exit_ ≥ 140° based on the angular measurements of the ED-ES system in their symptomatic ear (28). In addition, ears with α_exit_ between 120° and 140° were considered intermediate. Representative cases are presented in Figure 3. Hereafter, subgroups are collectively referred to as “ATVA subgroups” and independently referred to as “MD-120” (α_exit_ ≤ 120°), “MD-intermediate” (α_exit_ 120–140°), or “MD-140” (α_exit_ ≥ 140°). In the case of discordant ATVA subgroups between both ears from bilateral MD patients, we primarily stratified patients based on the presence of at least one MD-140 ear. After the exclusion of the MD-140 endotype, patients were stratified to the MD-120 subgroup if present. Otherwise, they were considered MD-intermediate.

The level of internal consistency of the MRI-based stratification process was tested by reassessing the MRI data from a randomly selected 20% of cases. The observer (LP) was blinded to the previous measurements when repeating the α_exit_ measurements and stratified the patients to either one of three ATVA subgroups (MD-120, MD-intermediate, MD-140) as aforedescribed. The intraobserver reliability of the ATVA subgroup allocation was determined using Cohen's kappa (κ) coefficient. A κ-value of 0.88 was determined, indicating excellent intraobserver agreement.

#### EH and perilymph signal intensity

The methods for evaluating EH and perilymphatic enhancement have been reported previously (31). Briefly, EH was scored as a blinded consensus reading by one head and neck radiologist (SH) and one PhD student (LP) on a 4-point scale for vestibular hydrops and a 3-point scale for cochlear hydrops, respectively. Quantitative measurements of the perilymph signal intensity were performed by one observer (LP) blinded to the clinical data. A freehand region of interest (ROI) was drawn on an axial section in the basal cochlear turn of both ears. An additional circular ROI of 0.6 mm^2^ was placed in the left middle cerebellar peduncle. The signal intensity ratio (SIR) of the basal cochlear turn to that of the middle cerebellar peduncle was calculated.

### Clinical data

Methods for clinical data collection have been reported previously (31). Briefly, clinical diagnoses were evaluated as a consensus reading by two otorhinolaryngologists (HB and CB), who were blinded to the MRI results, according to the latest AAO-HNS criteria (2). Patients who did not fulfill the clinical criteria for MD were assigned the clinical diagnosis of *other vertigo-associated pathology*, which was considered an umbrella term for other vertigo-associated diseases (e.g., vestibular neuritis and vestibular migraine) and non-classifiable cases. Data on symptoms and audiovestibular function tests were collected from electronic medical records. Age was defined as age at the time of MRI. Disease duration was calculated as the time elapsed from the first appearance of vertigo or hearing loss until MRI at our hospital. For hearing loss and vestibular function analysis, we selected the most recent pure-tone audiometry (PTA) and electronystagmography (ENG) seen from the moment of MRI with a maximum time interval of 1 year. We documented hearing function in the form of low and high Fletcher indexes (the average hearing loss at the frequencies 0.5–1.0–2.0 kHz and 1.0–2.0–4.0 kHz, respectively).

### Statistical analysis

Statistical analyses were performed using SPSS Statistics (version 28.0.1.0 and version 25.0., IBM, Chicago, Illinois, USA). The Kolmogorov–Smirnov test was used to test the data for normal distribution. Data are presented as median (min; max) or mean ± standard deviation. For binary variables, a chi-square test was performed if all category frequencies were >5; otherwise, Fisher's exact test was performed. Means and medians were compared by an independent *t*-test or Mann–Whitney *U*-test. A Fisher–Freeman–Halton test, one-way ANOVA, and Kruskal–Wallis *H*-test were used to compare binary variables, means, and medians among different ATVA subgroups. The level of significance was set at *P* < 0.05. In all contingency tables where significant differences were detected between the three groups, we performed *post-hoc* testing for pairwise comparison.

## Ethics

This institutional board-approved retrospective study was performed with a waiver of informed consent.

## Results

### Baseline characteristics

In total, 301 subjects were included in the final sample. The baseline characteristics of these subjects are summarized in Table 1. The most common clinical diagnoses of other VAP patients were vestibular migraine (17.5%), sudden sensorineural hearing loss (SSNHL) (15.8%), unspecified vertigo (15.8%), and vestibular neuritis (10.5%) (Table 2).

**Table 1:** Baseline characteristics of included patients (*n* = 301).

**Male/female, *n* (%)**	**126/175 (41.9/58.1)**
Mean age at MRI, *y*	54.8 ± 13.1
Mean age of onset, *y*	47.7 ± 13.9
Median disease duration, *y*	3.2 (0.0; 45.9)

MRI, magnetic resonance imaging.

**Table 2:** Diagnosis of other VAP patients (*n* = 114).

	***N* (%)**
BPPD	4 (3.5)
Cerebellar vertigo	1 (0.9)
Functional symptoms	2 (1.8)
**Hearing loss**	
Fluctuating low-frequency hearing loss	1 (0.9)
Low-frequency hearing loss	1 (0.9)
Presbycusis	1 (0.9)
Sudden SSNHL	18 (15.8)
Unspecified hearing loss	3 (2.6)
Hyperventilation	7 (6.1)
Inconclusive clinical diagnosis	7 (6.1)
Labyrinthitis (acute)	1 (0.9)
Labyrinthitis (chronic)	1 (0.9)
Mixed phenotype^*^	10 (8.8)
Orthostasis	1 (0.9)
Schwannoma	1 (0.9)
Tinnitus and aural fullness	1 (0.9)
Vascular loop	1 (0.9)
Vertigo, unspecified	18 (15.8)
Vestibular hypofunction and BPPD	1 (0.9)
Vestibular migraine	20 (17.5)
Vestibular neuritis	12 (10.5)
Vestibulopathy	2 (1.8)

BPPD, benign paroxysmal positional vertigo; SSNHL, sensorineural hearing loss.

^*^Mixed phenotype indicates multiple clinical diagnoses: BPPD, hyperventilation and/or vestibular migraine.

### Visibility of the ED-ES system

Among definite MD patients, the ED-ES system was visible bilaterally in 141 (75,4%) patients, unilaterally in 29 (15,5%) patients, and bilaterally deficient in 17 patients (9,1%). Non-visualization of the ED-ES system was significantly correlated with the symptomatic ears from MD patients (*P* = 0.038) and the presence of EH (*P* < 0.001). The latter was demonstrated in 192 (94.1%) symptomatic ears vs. 14 (8.2%) asymptomatic contralateral ears from definite MD patients.

Among other VAP patients, the ED-ES system was visible bilaterally in 108 (94.7%) patients, unilaterally in four (3.5%) patients, and bilaterally deficient in two (1.8%) patients. Other VAP patients with a deficient ED-ES system had a clinical diagnosis of vestibular migraine (*n* = 2), cerebellar vertigo (*n* = 1), hyperventilation-associated vertigo (*n* = 1), or unspecified vertigo (*n* = 1). Additionally, non-visualization of the ED-ES system was observed in two asymptomatic contralateral ears from two other VAP patients.

Based on these data, non-visualization of the ED-ES system showed a significant correlation with a clinical diagnosis of definite MD (*P* < 0.001, Table 3) with a positive predictive value of 87.5%.

**Table 3:** Visibility of the ED-ES system.

	**Definite MD (*n* = 204 symptomatic ears)**	**Other VAP (*n = 167 symptomatic ears)***	***P***
Visibility of the ED-ES system			<0.001
Visible, *n (%)*	162 (79.4)	161 (96.4)	
Non-visible, *n (%)*	42 (20.6)	6 (3.6)	

ATVA, angular trajectory of the vestibular aqueduct; deg, degrees; ED, endolymphatic duct; ES, endolymphatic sac; MD, Menière's disease; VAP, vertigo-associated pathology.

### Angular trajectory of the ED-ES system

The angular trajectory of the ED-ES system was evaluated in 114 definite MD patients (103 unilateral MD and 11 bilateral MD) and 96 other VAP patients (50 unilateral and 46 bilateral).

#### Definite MD vs. VAP

In general, the ED-ES system from symptomatic ears in definite MD patients demonstrated a larger α_exit_ compared with symptomatic ears from other VAP patients (*p* < 0.001; Table 4).

**Table 4:** Angular trajectory of the ED-ES system.

	**Definite MD**	**Other VAP**	***P***
Median α_exit_, *deg*	117.0 (88.3; 173.8)	109.5 (86.3; 156.3)	<0.001

α_exit_, exit angle of the ED-ES complex; deg, degrees; ED, endolymphatic duct; ES, endolymphatic sac; MD, Menière's disease; VAP, vertigo-associated pathology.

#### Clinical-radiological correlations in definite MD

Of the 114 included MD patients, 69 patients (60.5%) demonstrated an α_exit_ ≤ 120°, 29 patients (25.4%) had an α_exit_ between 120° and 140°, and the remaining 16 patients (14.0%) demonstrated an α_exit_ ≥ 140°. The clinical and radiological features of these patients are summarized in Table 5.

**Table 5:** Correlations between ATVA subgroups in definite MD patients (*n* = 114).

	**Data available, *n***	**MD-120 (*n* = 69)**	**MD-intermediate (*n* = 29)**	**MD-140 (*n* = 16)**	***P***
Female: male ratio	114	44:25	15:14	5:11	0.053
Unilateral: bilateral MD ratio	114	63:6	29:0	11:5	0.0045
Mean age at MRI, *y*	114	54.7 ± 13.2	59.2 ± 13.1	61.4 ± 10.5	0.144
Tinnitus, *n (%)*	83	39 (83.0)	24 (96.0)	10 (90.9)	0.121
Aural fullness, *n (%)*	64	33 (89.2)	17 (89.5)	8 (100)	1.000
Headache, *n (%)*	77	20 (44.4)	14 (58.3)	4 (50.0)	0.526
**Hearing loss**					
Mean age of onset, *y*	44	48.7 ± 14.6	56.8 ± 14.3	50.5 ± 18.8	0.495
Median disease duration, *y*	44	4.5 (0.0; 17.0)	5.7 (1.6; 12.9)	8.5 (0.3; 29.0)	0.522
Median low Fletcher, *dB*	97	48. (2; 108)	53 (13; 75)	45 (23; 120)	0.900
Median high Fletcher, *dB*	97	49 (5; 108)	52 (18; 75)	50 (28; 120)	0.977
**Vertigo**					
Mean age of onset, *y*	80	48.9 ± 14.2	50.8 ± 12.9	45.4 ± 11.6	0.483
Median disease duration, *y*	80	2.8 (0.1; 30.7)	4.9 (1.1; 11.0)	16.0 (0.4; 29.0)	0.006
Mean vertigo attack frequency, *nr per month*	70	6.3 ± 6.9	8.2 ± 10.8	5.0 ± 8.8	0.486
Drop attacks, *n (%)*	49	2 (6.3)	1 (11.1)	1 (12.5)	0.432
ENG hypofunction, *n (%)*	39	15 (55.5)	3 (42.9)	2 (40.0)	0.694
**EH**					
Cochlear EH grade	114	1 (0; 2)	1 (0; 2)	1 (0; 2)	0.409
Vestibular EH grade	114	2 (0; 3)	2 (0; 3)	2 (1; 3)	0.589
**Perilymph enhancement**					
Median SIR	114	1.45 (0.68; 5.0)	1.64 (0.71; 5.52)	1.48 (0.93; 3.66)	0.742

EH, endolymphatic hydrops; ENG, electronystagmography; MRI, magnetic resonance imaging; SIR, signal intensity ratio.

Among the 18 variables tested, two clinical variables demonstrated significant differences between ATVA subgroups. There was strong evidence for a different rate of bilateral clinical MD (*p* = 0.0045), with bilaterality being more prevalent among MD-140 patients compared with MD-120 and MD-intermediate patients (*post-hoc* pairwise analysis: *p* = 0.029 and *p* = 0.004). Within the MD-140 group, five patients had unilateral α_exit_ ≥ 140° and 11 patients had bilateral α_exit_ ≥ 140°, hereafter referred to as MD-140_uni_ and MD-140_bilat_, respectively. Bilateral MD was observed in two MD-140_uni_ patients and three MD-140_bilat_ patients (*p* = 1.000). There was further evidence for a longer history of vertigo for MD-140 patients compared with MD-120 patients (*p* = 0.006). Notably, the average age of onset of vertiginous symptoms did not differ between ATVA subgroups (*p* = 0.483). The initial analysis revealed a trend toward a different sex distribution among the three subgroups, with a female predominance in the MD-120 subgroup, a nearly balanced female:male ratio in the MD-intermediate subgroup, and a male predominance in the MD-140 subgroup. However, these results were just above the level of significance (*p* = 0.053). No significant differences were found regarding the onset or history of hearing loss, the presence or frequency of vertigo attacks, or the presence of tinnitus, aural fullness, migraineous symptoms, or drop attacks.

Pure-tone audiometry (PTA) was performed in 97 (85.1%) definite MD patients with an average time interval between MRI and PTA of 39 days (0; 365). The average low- and high-frequency hearing loss of all clinically affected ears was 48 (2; 120) dB and 50 (5; 120) dB, respectively. There were no significant differences regarding the average low- or high-frequency hearing loss between the ATVA subgroups (*p* = 0.900 and 0.977).

ENG was performed in 39 of 114 (34.2%) definite MD patients with an average time interval between MRI and ENG of 56.5 days (0; 328). Of these 39 patients, 20 (51.3%) demonstrated vestibular hypofunction in their symptomatic ear. The rate of vestibular hypofunction did not differ between the ATVA subgroups (*P* = 0.694).

In total, 106 (95.5%) patients demonstrated EH in their symptomatic ear. No significant differences were noted in the degree of cochlear or vestibular EH, or the intensity of perilymph enhancement between the ATVA subgroups (*p* = 0.409, *p* = 0.589, and *p* = 0.742). In unilateral MD patients, EH was noted in asymptomatic contralateral ears from two MD-120 patients, one MD-intermediate patient, and one MD-140 patient. Silent contralateral EH was not associated with a specific ATVA subtype (*p* = 0.560).

## Discussion

In the current study, we evaluated the appearance of the ED-ES system on 4 h-delayed Gd-enhanced 3T MRI in a spectrum of vertigo-associated disorders. We demonstrated that deficient visualization of the ED-ES system is associated with MD and EH and that, in cases of asymmetry between ears of MD patients, the symptomatic ear demonstrated poorer visualization of the ED-ES system compared with the asymptomatic ear. These findings are in accordance with previous authors investigating the visibility of the ED and ES in MD patients on non-contrast-enhanced 1.5 T MRI and support the hypothesis that obliteration of the ED-ES system may predispose to, or be associated with, the development of EH and MD (22, 32). We assume that a non-discernable ED-ES system could be explained by a reduced caliber of the VA, which is a well-described phenomenon in CT studies from MD patients (18, 33–35). A previous temporal bone study reported that the sizes of the ED and ES depend upon the size of the VA, which further supports this hypothesis (36). Although we cannot exclude that deficient visibility of the ED-ES system may also result from reduced vascularization or increased fibrotic changes in the surrounding connective tissue, this does not seem a likely explanation as previous histopathological studies revealed no differences in the perisaccular vascularization or degree of fibrosis between MD and healthy controls (37–39).

The morphology of the ED-ES system in other vertigo-associated diseases has scarcely been investigated. Leng et al. demonstrated poorer visibility of the ED-ES system in MD compared with vestibular migraine (VM) on non-contrast-enhanced MRI, though the authors reported that this finding was of low diagnostic value (40). In the present study, non-visualization of the ED-ES system was significantly associated with an MD diagnosis and only present in 5.3% of other VAP patients, which does not seem to diverge significantly from the reported prevalence in healthy controls (range 0–12.5%) (21, 22, 32). Therefore, lack of a visible ED-ES system may potentially serve as a discriminating radiological marker for MD with a high positive predictive value (88%). A visible ED-ES system, however, does not rule out an MD diagnosis.

The angular morphology of the VA/ED-ES system was first investigated by Eckhard et al. in 2019, and a custom-made open-source web application was subsequently developed (27, 41). In our experience, the use of the ATVA software on MRI was easily applicable and reproducible, as evidenced by the excellent intraobserver agreement (0.88). We performed angular measurements of the ED-ES system on 4 h-delayed 3D FLAIR images; an MRI technique that is increasingly used to detect EH and relies upon the selective enhancement of perilymph (42). Note that the ED and ES are membranous structures that are *not* surrounded by a perilymphatic space. They *are* however bounded by a relatively voluminous vascular network of supportive tissue (13). The enhancing structures in the region of the VA at Gd-enhanced FLAIR MRI therefore likely represent the periductal and perisaccular stroma, which we used as an indirect measure to delineate the ED-ES system in our study. Compared with other portions of the labyrinth, the ED-ES system harbors a relatively small volume of endolymph, which was not visible on our FLAIR sequence, probably due to insufficient spatial resolution.

According to previous studies from Bächinger et al., a radiological α_exit_ ≥ 140° of the ED-ES system corresponds to a hypoplastic (underdeveloped) ES that lacks an extraosseous portion, which has been suggested to be prenatally determined given its resemblance to the fetal configuration of the ED-ES system during gestational weeks 6 to 38 (29). In our cohort, the MD-140 endotype was present in 14.0% (16 from 114) of MD patients. This percentage is lower than the prevalence found in the MRI study from Bächinger et al. (17 from 72 patients, 23.6%), which may be explained by differences in sample sizes (29). We demonstrated that the MD-140 endotype is associated with bilateral clinical disease and a trend toward a male predominance, which is in accordance with previous studies (27, 29). Generally, MD symptoms manifest in the fourth decade of life (31). The presumed congenital origin of the MD-140 subtype raises the question of whether these patients would manifest symptoms earlier in life compared with the rest of the MD population. Compared with the other ATVA subgroups, MD-140 patients demonstrated a longer history of vertigo and an earlier disease onset, although the latter was not statistically significant. Notably, other authors have hypothesized that other epithelia in the inner ear may adjust and compensate for the early loss of ES function over a long period of time, which may explain the lack of a statistically significant earlier disease onset (43). These findings (longer disease duration, bilaterality, trend toward male predominance) may support the hypothesis from Bächinger et al. that genetic/developmental malformations of the ED-ES system underlie this MD subtype, although the multifactorial character of MD underlines the possibility that additional precipitating factors are necessary to elicit MD. Among the general MD population, familial clustering has been reported in 5–20% of cases, which also supports a genetic origin as a contributing factor in the etiology of MD (5). In the study from Bächinger et al., MD as well as isolated hearing loss/vertigo symptoms were significantly more prevalent in relatives of MD-140 patients compared with MD-120 patients (29). In our study, data on family medical history were not available.

The unpredictable clinical course of MD creates difficulties for patients and clinicians. One of the major concerns for unilateral MD patients is the progression to bilateral disease, which is associated with bilateral SSNHL and loss of vestibular function (44). In the case of bilateral affection, non-invasive therapy is preferred over ablative treatments with the intent to preserve as much inner ear function as possible (45). Similar reservations are relevant when considering destructive therapy in unilateral MD without knowing the probability of future bilaterality. However, identifying patients at risk for bilateral affliction is difficult due to the lack of a prognostic biomarker and delay of contralateral involvement, which may take up to 20 years or more to develop (46, 47). Bächinger et al. previously reported that, among MD-140 patients, conversion to bilateral MD is more common in patients with the MD-140_bilat_ endotype and can basically be excluded in MD-140_uni_ patients. Accordingly, based on their data, Bächinger et al. rendered MD-140_uni_ patients as the most suitable candidates for unilateral ablative therapy (29). However, in our study, bilateral clinical disease was observed in both MD-140_uni_ and MD-140_bilat_ patients. Although the sample sizes are small, we can conclude that a unilateral MD-140 endotype does not preclude the development of bilateral MD and we hypothesize that factors other than the ED-ES complex must be involved in its disease pathogenesis—a possibility that was also acknowledged by Bächinger et al. (30). The normal homeostasis of the inner ear is dependent on various regulatory mechanisms, such as fluid secretion and absorption, ionic transport, blood supply, and the integrity of the membranous labyrinth barrier system (48). MRI allows for the evaluation of the ED-ES system, EH as well as the permeability of the BLB, which is reflected in the degree of perilymphatic enhancement (10, 31, 49–51). There are scarce data on the morphology of the ED-ES system in relation to the severity of EH. Grosser et al. reported a correlation between the visibility/width of the VA on CT and the severity of cochlear EH on subtraction MR images (52). In addition, da Costa Monsanto et al. investigated histological specimens from MD patients and found that subjects with profound EH had smaller VA/ED-ES systems compared with patients with slight or moderate EH, although their findings were not statistically significant (20). To the best of our knowledge, the correlation between ATVA, EH, and BLB impairment has not yet been investigated. In our study, we did not observe a correlation between these MRI parameters.

The MD-120 subtype in our cohort was present in 61.3% of MD cases. For this group, we found no relevant clinical associations besides a trend toward a female preponderance. This finding seems to correspond to the gender distribution in the general MD population, in which a slight female preponderance has also been reported (6, 29, 53). According to previous studies, this MD subtype corresponds to a degenerative pathological process where the extraosseous portion of the ES epithelium showed pycnotic nuclei, shrunken or expelled cells, and fibrotic replacement (27). The degenerative ES endotype as described by Bächinger et al. is radiologically “diagnosed” by excluding the hypoplastic endotype. However, it cannot be differentiated from a *normal* ES, which also demonstrates an α_exit_ ≤ 120° on MRI (29). Bächinger et al. revealed an association between MD-120 and “silent” (asymptomatic) EH in the contralateral ear. In our study, we did not find such an association. However, the prevalence of silent contralateral EH was scarce (4%) in our cohort, and therefore, reliable conclusions regarding this putative association probably cannot be drawn from our data.

In total, 29 (26.1%) MD patients in our cohort demonstrated exit angles between 120° and 140°, which we referred to as “intermediate”. Bächinger et al. found intermediate angles in 21.7% of their cohort and excluded these patients from further analysis as they regarded the values inconclusive (i.e., not indicative of either a degenerative or hypoplastic ES endotype). The histopathological study from Eckhard et al. reported the presence of either ES degeneration or ES hypoplasia in *most* (95.8%) MD patients, but not *all* MD patients. Therefore, we chose not to exclude the MD-intermediate group as we aimed to evaluate clinical-radiological correlations from a broad perspective without the assumption regarding the associated ES pathologies. Nevertheless, the MD-intermediate group is a vaguely classified group, merely defined by the exclusion of ≤ 120° or ≥140° angles, that (in literature so far) does not correspond to a specific underlying pathology. The lack of significant findings in the MD-intermediate group was therefore not an unexpected result.

There are several limitations to our study. Despite our large study cohort, few patients with an MD-140 subtype (*n* = 16) were available for statistical analysis. A larger sample size is required to verify the reported radiological–clinical associations. In addition, data on family history were unavailable in our study. We could therefore not evaluate the aggregation of MD among relatives from MD-140 patients, which would further support the suggested genetic origin of this subtype. Third, the fact that our hospital is a referral center for vertigo may cause an underrepresentation of cochlear symptoms in our cohort, in particular tinnitus and aural fullness. Fourth, the retrospective nature of this study is prone to information bias, especially regarding the history of auditory symptoms, which may be subtle at the beginning of the disease and often overshadowed by debilitating vestibular symptoms. Prospective studies are needed to further investigate the radiological appearance of the ED-ES system in correlation with cochleovestibular symptoms and other clinical parameters, which could have a significant impact on our understanding of the disease, as well as the diagnosis and counseling of MD patients.

## Conclusion

In summary, our data corroborate morphological changes of the ED-ES system as a factor in the pathophysiology of MD. Additionally, we demonstrated that the MD-140 subtype is associated with bilateral clinical disease, a longer history of vertigo, and a trend toward a male predominance.

## Data availability statement

The original contributions presented in the study are included in the article/supplementary material, further inquiries can be directed to the corresponding author.

## Ethics statement

Ethical approval was not required for the study involving humans in accordance with the local legislation and institutional requirements. Written informed consent to participate in this study was not required from the participants or the participants' legal guardians/next of kin in accordance with the national legislation and the institutional requirements.

## Author contributions

LP, SH, and HB contributed to the conception and design of the manuscript. LP, SH, TV, MH, HB, and CB acquired the data. LP performed the statistical analysis and drafted the manuscript. All authors contributed to the article and approved the submitted version.
